# Characterization of PTPRG in Knockdown and Phosphatase-Inactive Mutant Mice and Substrate Trapping Analysis of PTPRG in Mammalian Cells

**DOI:** 10.1371/journal.pone.0045500

**Published:** 2012-09-20

**Authors:** Wandong Zhang, Katerina V. Savelieva, David T. Tran, Vladimir M. Pogorelov, Emily B. Cullinan, Kevin B. Baker, Kenneth A. Platt, Sean Hu, Indrani Rajan, Nianhua Xu, Thomas H. Lanthorn

**Affiliations:** Neuroscience Research, Lexicon Pharmaceuticals, Inc., The Woodlands, Texas, United States of America; Rutgers University, United States of America

## Abstract

Receptor tyrosine phosphatase gamma (PTPRG, or RPTPγ) is a mammalian receptor-like tyrosine phosphatase which is highly expressed in the nervous system as well as other tissues. Its function and biochemical characteristics remain largely unknown. We created a knockdown (KD) line of this gene in mouse by retroviral insertion that led to 98–99% reduction of RPTPγ gene expression. The knockdown mice displayed antidepressive-like behaviors in the tail-suspension test, confirming observations by Lamprianou et al. 2006. We investigated this phenotype in detail using multiple behavioral assays. To see if the antidepressive-like phenotype was due to the loss of phosphatase activity, we made a knock-in (KI) mouse in which a mutant, RPTPγ C1060S, replaced the wild type. We showed that human wild type RPTPγ protein, expressed and purified, demonstrated tyrosine phosphatase activity, and that the RPTPγ C1060S mutant was completely inactive. Phenotypic analysis showed that the KI mice also displayed some antidepressive-like phenotype. These results lead to a hypothesis that an RPTPγ inhibitor could be a potential treatment for human depressive disorders. In an effort to identify a natural substrate of RPTPγ for use in an assay for identifying inhibitors, “substrate trapping” mutants (C1060S, or D1028A) were studied in binding assays. Expressed in HEK293 cells, these mutant RPTPγs retained a phosphorylated tyrosine residue, whereas similarly expressed wild type RPTPγ did not. This suggested that wild type RPTPγ might auto-dephosphorylate which was confirmed by an *in vitro* dephosphorylation experiment. Using truncation and mutagenesis studies, we mapped the auto-dephosphorylation to the Y1307 residue in the D2 domain. This novel discovery provides a potential natural substrate peptide for drug screening assays, and also reveals a potential functional regulatory site for RPTPγ. Additional investigation of RPTPγ activity and regulation may lead to a better understanding of the biochemical underpinnings of human depression.

## Introduction

Protein tyrosine phosphatases (PTPs) are thought to counteract the action of tyrosine kinases and are necessary for the finely regulated balance of tyrosine phosphorylation in cell signaling that is critical for cell growth, differentiation and proliferation. However, while the roles and mechanisms of tyrosine kinases are well characterized, the functions of tyrosine phosphatases are less known. PTPs can be classified into two groups: cytoplasmic and transmembrane receptor-like. The transmembrane receptor-like protein tyrosine phosphatase (RPTP) family includes RPTPγ that, along with its close homolog RPTPζ, forms a subfamily of RPTPs [1]. RPTPs have extracellular domains resembling those of adhesion molecules and possess functions in cell adhesion, homophilic binding, and outgrowth promotion [2], [3], [4], [5], [6]. RPTPγ and RPTPζ bind differentially to homologous contactins, which are neuronal cell adhesion molecules, via their respective extracellular domains [7], [8]. Genetic analyses in drosophila and C. *elegans* support roles of RPTPs in axonal guidance during development and synapse formation which involves an adhesion-like function [5], [9], [10], [11].

The intracellular domains of RPTPs consist of one or two tyrosine phosphatase domains, of which at least one possesses active tyrosine phosphatase activity. It is thought that RPTPs regulate protein tyrosine phosphorylation states and this hypothesis is supported by some lines of evidence. For example, RPTPs such as RPTPα, RPTPε, and RPTPζ, have been shown to regulate ion channels by regulating their phosphorylation [12], [13], [14]. In particular, activation of the maxi-anion channel involves protein dephosphorylation mediated by PTPs, including RPTPζ in mouse fibroblasts [15]. RPTPζ efficiently dephosphorylates TrkA receptor and attenuates NGF-dependent neurite outgrowth in PC12 cell [16].

Unlike receptor tyrosine kinases, the detailed mechanisms underlying RPTP-mediated extracellular binding or adhesion and phosphatase-mediated cell signaling are less well characterized. Fewer extracellular binding partners and fewer physiological substrates have been found to date. To investigate the role of the phosphatase domain and to identify physiological substrates, “substrate-trapping” mutations were invented and successfully employed [17], . In a trapping mutant, the phosphatase domain is mutated to render a catalytically-inactive site that leads to more stabilized binding of the otherwise transiently bound substrate.

RPTPγ has been widely studied as a tumor suppressor [e.g., 20, 21] which might be related to its negative regulation of tyrosine phosphorylation events, and substrates have been suggested. For example, the intracellular domain of RPTPγ directly interacts with BCR/ABL and CRKL and may regulate their phosphorylation status in HEK293 cells [22]. RPTPγ is abundant in the nervous system and its expression has been examined in detail using promoter-driven β-galactosidase [23]. It was shown to be expressed in both embryonic and adult brain, and in various sensory organs, but not in astrocytes [23]. It is interesting that RPTPζ may have complementary expression to that of RPTPγ. For example, in the central nervous system RPTPζ is strongly expressed in glial cells, but only in a subset of neurons [24], [25]. An RPTPγ null mouse line revealed that it can be spared for normal development and null mice exhibited only a few minor behavioral phenotypes [23].

In our current studies, we created a knockdown mouse genetic line with 98–99% reduction of RPTPγ gene expression, and conducted intensive neurobehavioral characterization of this mutant which revealed an antidepressive-like phenotype. To see if the antidepressive-like phenotype was due to the loss of phosphatase activity, we also made a knock-in (KI) mouse in which an inactive mutant phosphatase, RPTPγ C1060S, replaced wild type RPTPγ. The phenotypic analysis of the KI mice revealed that they also displayed some antidepressive-like phenotype. These results lead to a hypothesis that an RPTPγ inhibitor will be a potential treatment for human depressive disorders. Using substrate trapping mutants, with the aim to identify physiological substrates, we discovered that RPTPγ auto-dephosphorylates in HEK293 cells, and further went on to identify the dephosphorylation site.

## Materials and Methods

### Generation of Ptprg Knockdown Mutant Mice by Retroviral Insertion Gene Trap

**Figure 1:**
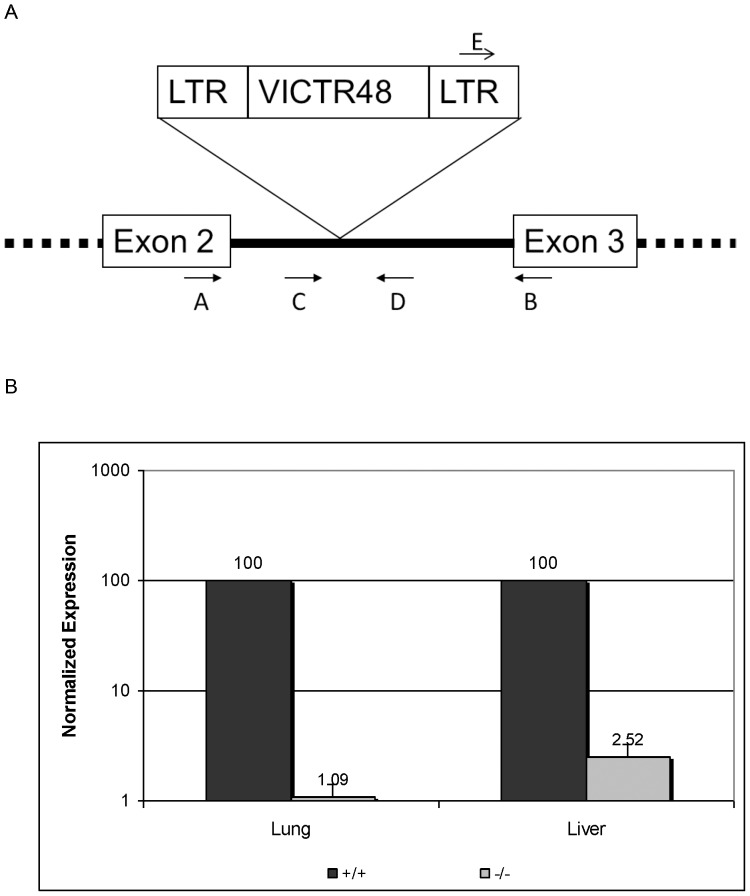
Generation of knockdown mice. A. Gene trap disruption of the Ptprg gene locus. Genotyping and RT-PCR/QRT-PCR primers are indicated by arrows, as described in the methods. Primers in Exons 2 and 3 are used for RT-PCR and QRT-PCR to assay the endogenous transcript in wildtype and mutant mice. Primers in the LTR and intron (solid line) are used to genotype the wildtype and mutant mice. B. QRT-PCR analysis of wildtype (+/+) and Ptprg knockdown (−/−) mice. Lung and liver tissues were analyzed from one +/+ and three −/− knockdown mice. Logarithmic scale.

**Figure 2:**
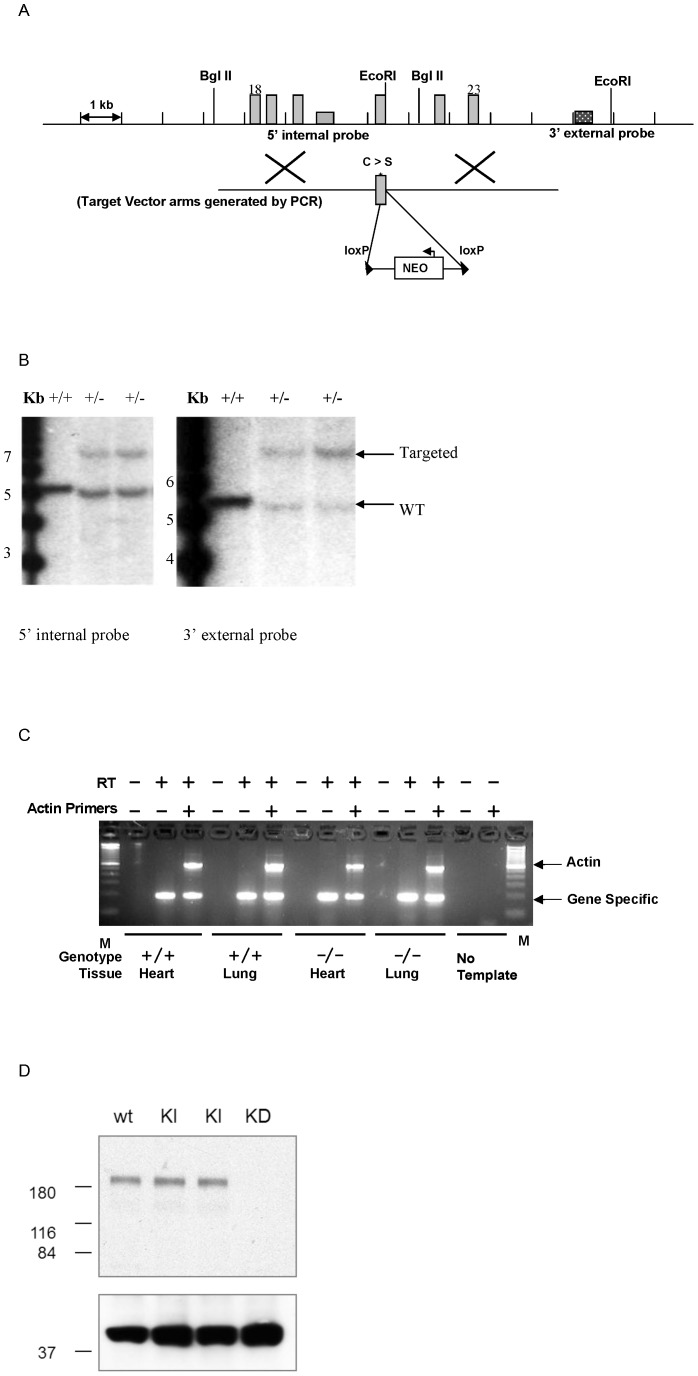
Introduction of a Ptprg point mutation. A, Targeting strategy used to introduce the point mutation into the Ptprg locus. Homologous recombination (represented by X) between the targeting vector and the Ptprg gene results in the replacement of a G residue with a C resulting in a Cysteine to Serine amino acid change. The LoxP flanked Neo cassette is excised during the chimeras breeding step. B, Southern hybridization indicating proper gene targeting in the embryonic stem cell clones. Lex represents untransfected embryonic stem cell DNA. C. RT-PCR sequence confirmation of the point mutation. Each sample includes a negative control without Reverse Transcriptase, an assay with Ptprg primers alone, and an assay that includes an internal positive control for Actin. D. western blotting. In the top panel, anti-RPTPγ antibody (9E3) detected a band slightly larger than 180 kDa in the wild type and knockin, but not in the knockdown. wt: wild type. KI: knockin. KD: knockdown (gene trap disruption of the Ptprg gene locus). Proteins loaded were 30 micrograms of proteins as membrane preparation from mouse brain without cerebellum. The bottom panel is a loading control showing a band slightly larger than 37 kDa which reacted nonspecifically to anti-RPTPγ (LGI13) and is present in all four samples. The molecular weight (kilodalton) is shown on the left.

Receptor tyrosine phosphatase gamma is encoded by Ptprg gene in mice. The Ptprg mutant mice were generated by gene trapping using OmniBank™ 129S5/SvEvBrd ES cell clone OST142086 as described in [26]. Identification of trapped genes by using OmniBank Sequence Tags (OSTs) and characterization of retroviral gene-trap vector insertion points were as described in [26], [27], [28], [29] (figure 1A). All mice were of mixed genetic background (129SvEvBrd and C57BL/6J-*Tyr* ^c-Brd^). RT-PCR was performed on RNA extracted from lung and liver of Ptprg mutant mice and a wildtype littermate using Ptprg specific primers (A: 5′-GCT TCC CGG CAT TGA CTG AAG GCT ATG TGG-3′, and B: 5′-AAC GCG AGC ATG GTG GTC TAA AAT GTC TAT-3′) that are from the second exon and from the third exon and flank the retroviral insertion site (figure 1A). This yields a PCR product of 209 bp in both mutant and wild type, and the PCR product is at much reduced levels in the mutant compared to the wild type (data not shown). This suggests that the retroviral insertion led to significant knockdown of the Ptprg gene expression. The reduction of Ptprg transcript was measured by QRT-PCR in the lung and liver of three mutant mice, using the same primers. GAPDH (primers 5′-ACA GTC AAG GCC GAG AAT and 5′-GGA GAT GAT GAC CCG TTT GG-3′) was included as an internal control (figure 1B). The QRT-PCR showed that knockdown is nearly complete and there is only about 1.1–2.5% of Ptprg transcript existed in the mutant lines. In Western blot analysis protein expression in this mutant line was undetected which again confirmed this knockdown line is nearly comparable to a null line (figure 2D).

Mice were genotyped by PCR. The mutated allele yields a PCR product of 277 bp using primers, E: 5′-AAA TGG CGT TAC TTA AGC TAG CTT GC-3′ in the LTR of the vector and primer D: 5′-TCA CAT CTG AGA CTA CCG TGT TC-3′ in the intron of Ptprg (figure 1A). The wildtype allele yields a PCR product of 276 bp using primers from the intron of Ptprg, C: 5′-AGG ACT GGT GGT AAG AAA CTA TT-3′ and D: 5′-TCA CAT CTG AGA CTA CCG TGT TC-3′ (figure 1A; data not shown).

### Generation of Ptprg Knockin Point Mutant Mice

The Ptprg point mutant targeting vector was generated by PCR using the lambda KOS genomic clone previously isolated. The 5′ arm containing terminal Sac II and Eco RI sites was generated using primers PtprgPM-1 (5′-GAA TTC TGA CCC TAC CTG ***C***AG TGC ACC AAC AC-3′) and PtprgPM-2 (5′-CCG CGG ATT AAA TGT GGC ACC ATC ATA CC-3′). The 4144 bp 5′ arm containing the G to C point mutation in exon 21 (the designed point mutation is indicated by the bold, italic underlined nucleotide that results in changing a Cysteine residue to Serine). A 273 bp flanking genomic region containing terminal Eco RI and Sfi I sites was amplified using primers PtprgPM-3 (5′-GAA TTC GGA GGG GGA TGA CTG G-3′) and PtprgPM-4 (5′-GGC CGC TAT GGC CAC AAT TCC CGT ACA GTA GTA GG-3′). The 3964 bp 3′ arm containing terminal Sfi I and Kpn I sites was amplified using primers PtprgPM-5 (5′-GGC CAG CGA GGC CGG GCT CAG CCT AAT AAA ATT TGA AC-3′) and PtprgPM-6 (5′-GGT ACC TCT CTC CAG CTG CGT TAC GAC CAC T-3′). To complete the targeting vector, these 3 fragments along with a 2.2 Kb LoxP flanked PGK-Neo cassette with terminal Sfi I ends were ligated together and inserted into a pKO Scrambler vector cut with Sac II and Kpn I (figure 2A). The Not I linearized targeting vector was electroporated into Protamine Cre 129S4/SvJae ES cells [30] resulting in selection cassette excision in the male germline. G418/FIAU resistant ES cell clones were isolated, and correctly targeted clones were identified and confirmed by Southern analysis using a 375 bp 5′ internal probe, generated by PCR using primers (5′-GAG CTC AGA CTG GCC TCT AAC-3′) and 5′-CAG AAG TCC TTG GGA TAC TCT-3′) and a 302 bp 3′ external probe (3/4) previously mentioned. Southern analysis using 5′ internal probe 5/6 detected a 5.0 Kb wild type band and a 7.2 Kb mutant band in Bgl II digested genomic DNA while 3′ external probe 3/4 detected a 5.5 Kb wild type band and 7.7 Kb mutant band in EcoRI digested genomic DNA (figure 2B). Targeted 129S4/SvJae ES cell clones were identified and microinjected into C57BL/6J-*Tyr* ^c-Brd^ blastocysts to generate chimeric animals which were bred to C57BL/6J-*Tyr* ^c-Brd^ females, and the resulting heterozygous offspring (with selection cassettes removed) were interbred to produce homozygous Ptprg point mutant mice.

Mice were genotyped by PCR. The wildtype allele yields a PCR product of 532 bp using primers within exon 21 and the downstream intron, 5′- GTC AGA ATG AGC GGA CGG TGA-3′ and 5′- TAT TAT CAT TTA CAG GA TCC C-3′. The mutated allele yields a PCR product of 626 bp from these same primers. The expression of the point mutation was confirmed in heart and lung by RT-PCR using primer 5′-CAG AAT GAG CGG ACG GTG ATC CAG T-3′ and 5′-GCT GTC TAT TAC AAT GTA GGT GCC TGT CC-3′ (figure 2C). Sequence analysis of the PCR product confirmed the change from G to C in the mutant homozygotes (not shown), which encodes a RPTPγ protein with C1060S mutation.

Western blotting confirmed the expression of mutant RPTPγ C1060S in knockin mice at a level comparable to that of wild type protein in wild type mice (figure 2D).

### Animals

The genetic manipulations were generated in ES cells derived from 129S5/SvEvBrd strain (knock-down) or 129S4/SvJae strain (knock-in), and the targeted ES cell clone was microinjected into albino C57BL/6J-*Tyr* ^c-Brd^ blastocysts for chimera production. Confirmed heterozygotes produced from chimera breeding were used for heterozygote interbreeding to generate homozygotes, heterozygotes and wild type controls that were used for experimental testing. Thus all animals used for studies, including wild type controls, were F2 (2^nd^ generation of the heterozygote interbreeding) cohort mates bred in a mixed (C57BL/6J-*Tyr* ^c-Brd^ × 129S) genetic background at Lexicon Pharmaceuticals. All mice were maintained under a standard light/dark cycle from 7 am to 7 pm. They were housed in groups of five in 30 cm x20 cm x20 cm acrylic cages with food and water freely available. Lexicon’s albino C57BL/6J-*Tyr* ^c-Brd^ strain is identical to the commercially available C57 non-albino strain (C57BL/6J from Taconic Farms, Inc.). The two C57 strains differ only by the spontaneous mutation that arose in the c locus, thus resulting in albinism. The detailed description of the albino strain can be found in MGI database (Phenotypic Allele Detail, C57/BL6J-Tyrc-Brd http://www.informatics.jax.org/javawi2/servlet/WIFetch?page=alleleDetail&key=43586). An albino strain was originally chosen as a background strain for all Lexicon Pharmaceuticals knockout mice because chimeras derived from albino embryos allow for a more accurate measurement of chimerism than non-albino black strain.

All the work with mice has been conducted according to the protocols approved by IACUC (Institutional Animal Care and Use Committee) of Lexicon Pharmaceuticals, Inc.

**Figure 3:**
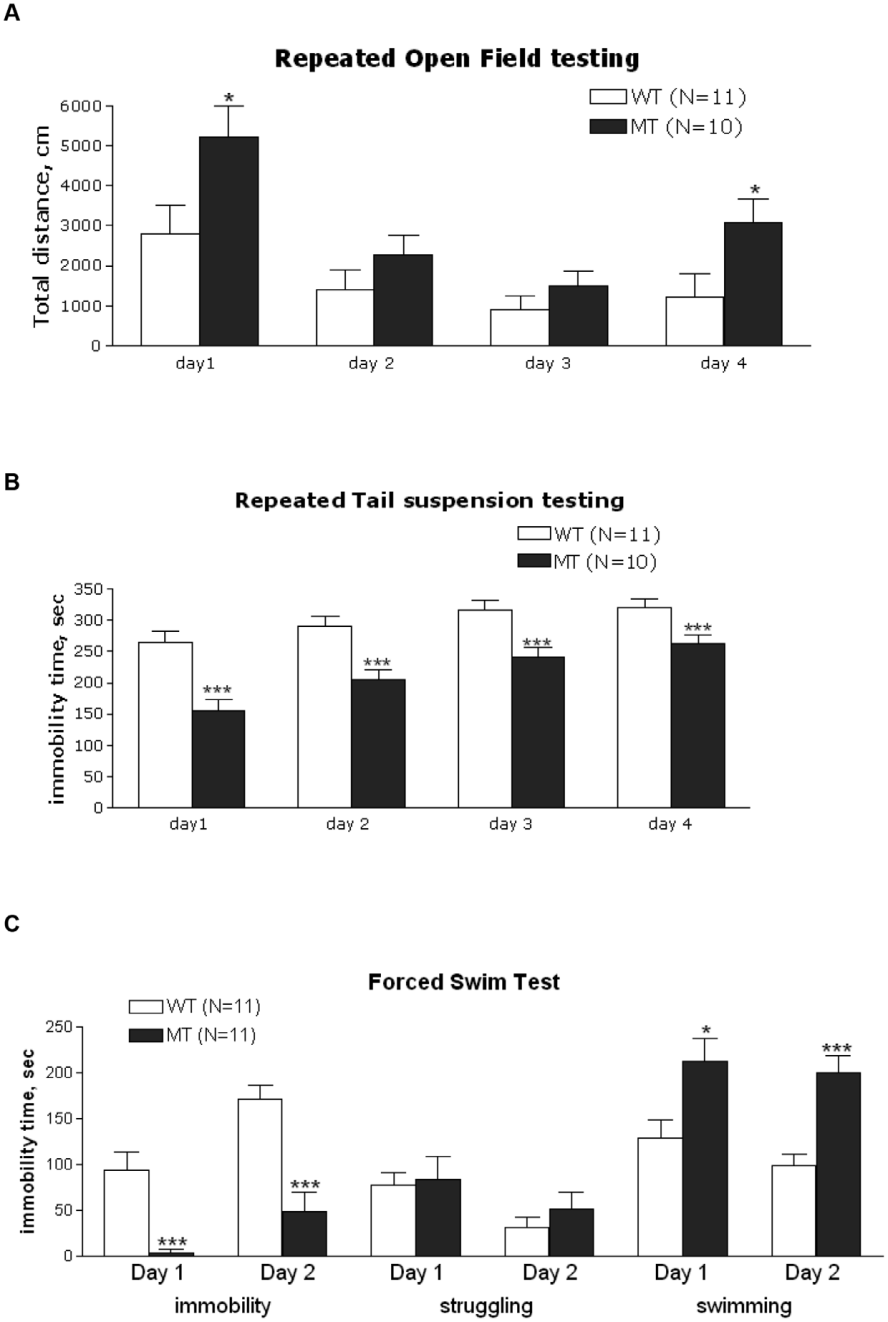
Behavioral analysis on the wild type (WT) and knockdown mutant mice (MT). See details in the text. A and B. Repeated open field (A) and tail suspension tests (B) on the same animals. * - P<0.05, *** - P<0.001, compared to WT on the same day. C. Immobility time in the forced swim test. * - P<0.05, *** - P<0.001, compared to WT for the same measure.

### Behavior Assays

Mice were tested in a standardized behavioral phenotyping battery. A comprehensive phenotypic analysis (including a subset of behavioral tests derived from the Irwin screen) revealed no notable abnormalities across a wide range of behaviors as well as assays for cardiac, immune system, endocrine, and ophthalmic function. The description of phenotyping battery and methods not described in the main text is available in our prior publications [31], [32].

#### Locomotor activity

Locomotor and exploratory behaviors were recorded with twelve Digiscan open field (OF) apparatus and Versamax software, v.4.00–127E (Accuscan Instruments, Inc., Columbus, OH). A large arena (42 cm x 42 cm) with infrared beams at three different levels was used to record horizontal locomotor activity (total distance), vertical movement (rearing), and investigation into the 4 holes in the floor of the open-field (hole poke). Two florescent lamps positioned over each chamber provided light levels of 800 lux in the center of each open field arena. Each animal was placed in the center of the open field and its activity was measured for 20 minutes. The total distance traveled (cm), vertical movement number (rearing), number of hole pokes, time spent in the center of the OF (time-in-center), distance traveled in the center of the OF (center distance), and center/total distance ratio over the intervals were recorded using Versadat program, v.2.70–127E (Accuscan Instruments). The OF center area measured 20 cm x 20 cm.

**Figure 4:**
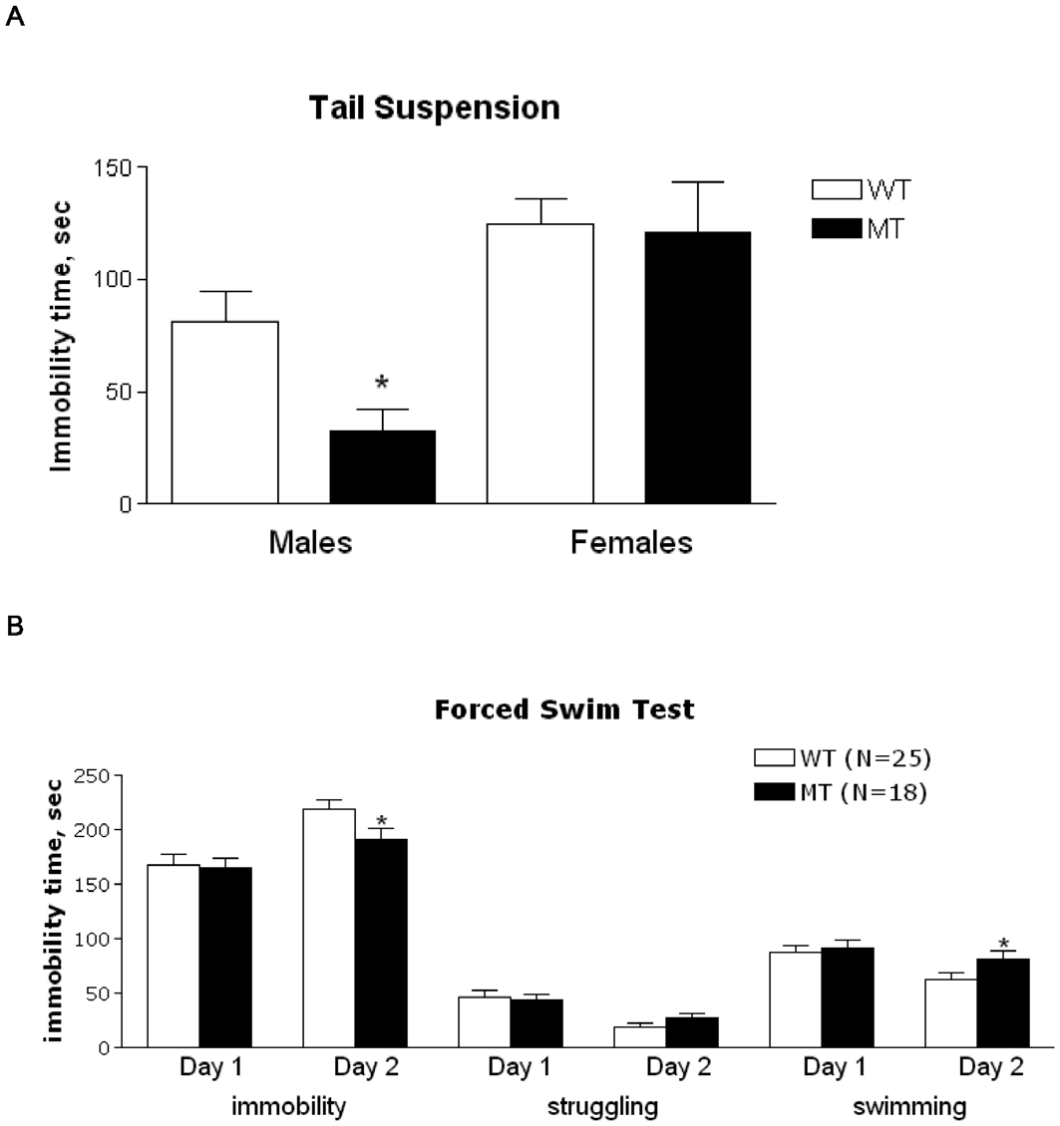
Behavioral analysis on the wild type (WT) and knockin mutant mice (MT). See details in the text. A. Immobility time in the tail suspension test. Male: WT, n = 8, MT, N = 7; Female: WT, n = 15, MT, n = 10. * - P<0.05, compared to WT of the same sex. B. Immobility time in the forced swim test. * - P<0.05, compared to WT for the same measure.

#### Tail suspension test (TS)

The TS test was conducted using 8 chambers (PHM-300TSS Mouse Tail Suspension System, Med Associates, Georgia, VT). Mice were securely fastened with medical adhesive tape by the tip (∼1.0–1.5 cm) of the tail to a metal hook (8.5 cm × 0.2 cm) and suspended above the floor in a visually isolated cubicle. Mobility was recorded by a linear load cell, load cell amplifier, and filter. Immobility was defined as the area under the curve using threshold = 2, gain = 4, and resolution = 100 ms. The duration of immobility over a single 6-min session was recorded for each mouse. For one cohort the test was repeated over four days. Animals that climbed their tails during testing on any of the test days were excluded from data analysis.

#### Forced swim test (FST)

The FST chamber was a 2000 ml Pyrex beaker with a diameter of 14 cm filled with deionized water (25°C) to a depth of 14 cm. A black Plexiglas chamber (30 cm x 28 cm x 20 cm) surrounded each beaker. The front and top of the chambers were open to allow for video recording. To optimize contrast, a black background was used for white mice, and a white background was used for agouti and black mice. With respect to the forced swim test, our in-house experience showed that measuring immobility for one six-minute session (standard mouse one-day protocol) is not sufficient to detect an antidepressant-like effect for certain antidepressants. We therefore employed a 2-day protocol similar to that commonly used for rats [33], [34] and found this to enhance the sensitivity of the test [32], [35].

On the first testing day mice swam for 15 minutes (pre-test session). Twenty four hours later, mice were exposed to the same experimental conditions for 5 minutes (test session). The water in the beaker was changed between each animal. The data was analyzed with image analysis software, VideoTrack Quantization (ViewPoint Life Sciences, Montreal, QC, Canada) and immobility and struggling were quantified for the duration of the first 5 minutes during pre-test (so that we also have data comparable to the standard one-day mouse FST) and all 5 minutes during the test session. Immobility was defined as minimal movements required for a mouse to keep its head above the water. Struggling was defined as vigorous movements with forepaws breaking the water. The same thresholds were set for all animals (<450 was considered immobility; >785 was counted as struggle). Swimming was defined in the program as activity measuring between thresholds 450 and 785.

**Figure 5:**
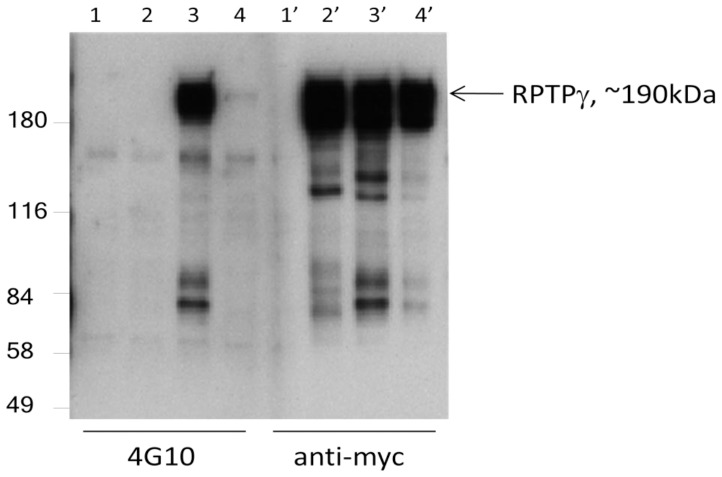
RPTPγ substrate-trapping mutants in HEK293 cells. Phosphotyrosine-containing protein of about 190 kDa size “trapped” by RPTPγ C1060S or RPTPγ D1028A is RPTPγ. Recombinant wild type RPTPγ and substrate-trapped mutants were immunoprecipitated from transiently transfected HEK293F lysate with anti-myc mAb. Lanes 1 and 1′were IP product from pCDNA3 transfected cells, 2 and 2′ were from wild type RPTPγ transfected cells, 3 and 3′ were from RPTPγ C1060S transfected cells and 4 and 4′ were from RPTPγ D1060A transfected cells. Lane 1–4 were western reacted with anti-phosphotyrosine 4G10 mAb and lanes 1′-4′ were with anti-myc mAb.

**Figure 6:**
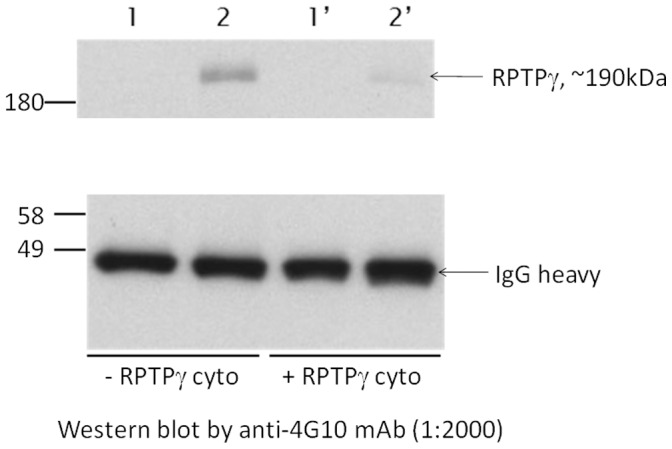
RPTPγ dephosphorylated itself in vitro. Wild type RPTPγ or RPTPγ C1060S plasmids were transiently transfected into HEK293F and isolated from lysates with anti c-myc/protein G sepharoses. RPTPγ wild type or C1060S on the protein G sepharose was denatured in 8 M urea to linearized protein as substrates for reaction following. The Purified RPTPγ wild type, C1060s sepharose beads was then incubated with recombinant, active purified RPTPγ cyto enzymes in assay buffer with urea at a final concentration of 0.375 M. Lanes 1 and 2 are RPTPγ WT and RPTPγ C1060S on protein G sepharose in a mock reaction without phosphatase in the same buffer, and lanes 1′and 2′ are RPTPγ wild type and RPTPγ C1060S reacted with RPTPγ cytoplasmic region as phosphatase. Samples were subjected to western blot analysis using anti-phosphotyrosine 4G10 mAb. The densitometry of each band was determined to estimate the extent of removal of phosphotyrosine on the protein. The reacted IgG heavy chain bands were used as internal control to ensure equal loading of samples. It is estimated by densitometry that 75% of phosphotyrosine from RPTPC1060S was removed by addition of RPTPγ enzyme.

### Data Analysis

Data are presented as individual data points or as means +/− SEM. The Statistica 8.0 software package (StatSoft, Inc., Tulsa, OK) was used to determine significant differences between groups. The data were analyzed with one-way ANOVA with genotype and sex (where both sexes were tested) as main effects. If no interactions between genotype and sex were observed, additional analyses employed unpaired, two-tailed t-tests (with the Welch’s correction where a significant difference in variance was detected). The data for OF and repeated tail suspension tests was analyzed using repeated measures ANOVA with genotype and sex as main effects, and test interval or day as a repeated measure.

### DNA Plasmids and Site-directed Mutagenesis

For functional study of RPTPγ protein, cDNA sequence encoding human receptor tyrosine phosphatase gamma-B isoform with c-myc and polyhistidine epitopes at carboxyl terminus was created in pCDNA3.1 (+) expression plasmid (pCDNA3.1+/RPTPγ mychis) expressing a B form of full length RPTPγ (1–1445 aa. without the 29 aa. specific for A form, NP_002832) using standard molecular biology techniques. The final constructs were confirmed by sequencing. “Substrate-trap” RPTPγ mutants, were generated by performing site-directed mutagenesis (QuikChange site-directed mutagenesis kit, Stratagene, San Diego, CA) directly on parental pCDNA 3.1 (+)/RPTPγ myc-his plasmid to change Cysteine at position 1061 to Serine or Aspartic acid at position 1028 to Alanine. The resulting mutant constructs, RPTPγ C1061S and RPTPγ D1028A, respectively, were sequenced to confirm that no other mutations had been introduced. To map the autodephosphorylation region, pCDNA 3.1 (+)/RPTPγ myc-his plasmid was truncated to produce a RPTPγ protein with D2 domain deleted (aa. 1–1127 of NP_002832). To further map the auto-dephosphorylation site eleven tyrosine residues in D2 domain were individually mutated to phenylalanine by PCR from parental full length RPTPγ pCDNA3.1 construct (figure S1). For in vitro assay of phosphatase activity, cytosolic region containing D1 and D2 domains (aa. 790–1445 of NP 002832) was cloned into pET24 vector (pET24/mycHisRPTPγ-cyto) and expressed and purified from E. coli as described in Appiah et al. [36]. In addition, to confirm the impairment of phosphatase activity in C1060S and D1028A mutants, cytosolic region containing catalytic domain D1 (aa. 790–1127 of NP002832) from wildtype or mutants was subcloned into pGEX5x-1 expression vector to obtain pGEX5x-1/RPTPγcyto D1.

**Figure 7:**
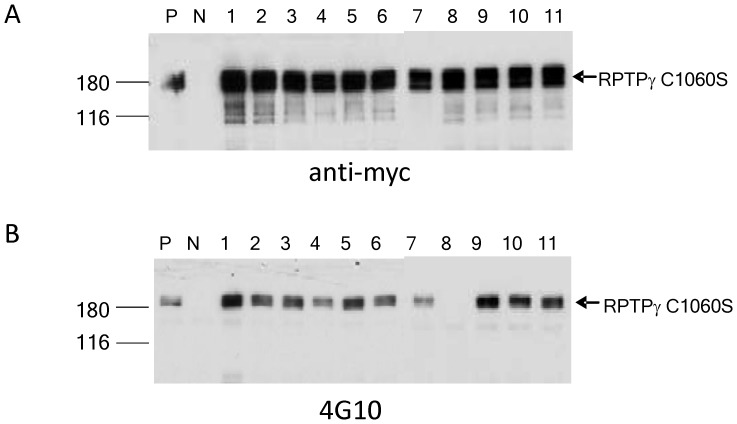
Identification of tyrosine residue at amino acid 1307 (Y1307) as auto-dephosphorylation site. There are a total of eleven tyrosine residues that can be found in D2 domain (figure S1). Each of these tyrosine residue was individually mutated to phenylalanine by PCR on the parental plasmid pCDNA3.1 RPTPγ C1060S. We then performed transfection of pcDNA3.1 RPTPγ C1060S (lanes “P” in panels A and B) and the 11 different pcDNA3.1 RPTPγ C1060S/Y->F mutants (lanes1 to 11 in panels A and B) to HEK293 cells. Lane “N” is vector control only. The immunoprecipitates by anti-myc were loaded to SDA-PAGE and subjected to western blot by anti-myc (A) and anti-4G10 (B). The results revealed that all mutants but RPTPγ C1060S/Y1307F (lane 8 in panel B) contained tyrosine-phosphorylation reacted to antibody 4G10 (B). We concluded that Y1307 is the tyrosine residue that was phosphorylated, by activity of an unknown kinase, and then dephosphorylated by active RPTPγ.

### Antibody Production and Purification

RPTPgamma cytoplasmic region was expressed from E. coli transformed with plasmid pET24 mycHisRPTPγ-cyto. The expressed protein was partially soluble and was partially purifed by nickel resin. The partially purified protein was run on preparative SDS-PAGE and RPTPγ cyto (aa. 790–1445 of NP 002832) was visualized by staining of 2 M KCl. The protein was excised from gel and the protein was electroeluted from gel piece to obtain highly purified protein. The purified protein was dialysed against saline and was used to immunize rabbit for polyclonal sera (LGI13) and Ptprg knockdown mutant mice for mouse polyclonal sera and monoclonal antibody (9E3).

### Expression and Functional Analysis of RPTPγ Mychis “Trapping Mutant”

Transient transfection of RPTPγ and substrate-trap mutants in HEK293F cells was performed with Lipofectamine 2000 reagents (Invitrogen, Carlsbad, CA). Transiently transfected cells were washed once with cold PBS, and incubated with 12 ml of Versene (Invitrogen) they started to detach from plates. Cells were gently pipetted up and down to disperse any clumps, transferred to 15 ml conical tubes and spin down at 2500 rpm for 3 minutes. The supernatant was removed and cell pellet was resuspended cold 7 ml of Lysis buffer (50 mM Tris-HCl, pH 7.5, 150 mM NaCl, 1 mM EDTA, 1% NP-40, 0.2 mM orthovandate, 1 × protease inhibitor (-EDTA). Centrifugation cleared lysate was transferred to new tube and small aliquot (5 micrograms of proteins) was used for western blot analysis was done to confirm expression of recombinant proteins.

**Figure 8:**
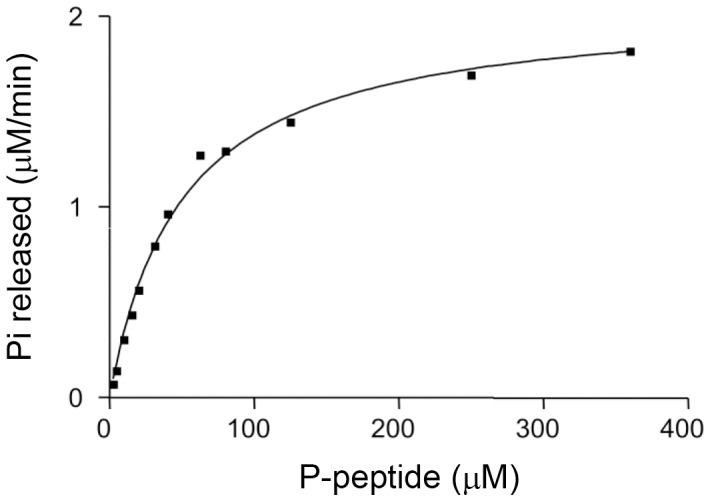
Determination of Km of phosphatase RPTPγ on peptide substrate ATQDD(pY)VLEVR (derived from peptide adjacent to Y1307). Figure here shows Pi released (microM per minute) was plotted against phospho-peptide in a reaction in which Vmax was 2.1 µM/mim/14 nM RPTPγ and Km is 49.6 µM, using one site nonlinear binding algorithm in Graphpad Prism.

To immunoprecipitate (IP) RPTPγ, RPTPγ C1061S or RPTPγ D1028A recombinant protein from HEK293F lysate for “substrate-trapped” study, lysate containing 100 micrograms of protein was pre-cleared by incubating lysate with Protein G sepharose beads (ThermoScientific) for one hour at 4°C with gentle end-over-end inversion to remove non-specific absorption to protein G beads, and collected by centrifugation at 12,000 rpm for 20 seconds at 4°C. RPTPγ specific polyclonal antibody 9E3 or monoclonal anti-c-myc antibody (Sigma Aldrich) was added at 1∶100 v/v to immunocapture RPTPγ, RPTPγ C1061S or RPTPγ D1028A recombinant protein from lysate and incubated for 1 hour with gentle end-over-end inversion. To precipitate RPTPγ, RPTPγ C1061S or RPTPγ D1028A recombinant protein-antibody complex, 50 ul of pre-washed protein G sepharose beads was added to each sample, incubated for 2 hour at 4°C with gentle end-over-end inversion. Precipitates were collected by centrifugation, washed 3 times with cold Lysis buffer at 10 minutes per wash, boiled in 60 ul of 1.5 × Tris-glycine SDS/5% β-mercaptoethanol loading buffer for 5 min, and 20 ul of each sample was loaded onto pre-cast Tris-Glycine SDS-PAGE gel for western blot analysis. 4 G10 monoclonal antibody (Upstate) specific for phosphor-tyrosine was used in western blots.

### RPTPγ Phosphatase Assays

To obtain RPTPγ C1060S and D1028A mutants and analyze their phosphatase activities, pGEX5x-1 plasmid containing RPTPγ cyto D1, RPTPγ cyto D1 C1061S or RPTPγ cyto D1 D1028A were transformed into BL21 DE3 and 1 ml of overnight culture was seeded into 100 ml of LB media containing 100 ug/ml ampicillin. Culture was shaken at 250 rpm at 30°C until OD600 0.5, then induced with 0.2 mM isopropyl-1-thio-β-D galactopyranoside (IPTG) for 4 hours at 30°C. Bacteria cultures were centrifuged and resuspended in cold Lysis buffer (50 mM Tris-HCl pH 8.0, 1 mM EDTA, 50 ug/ml DNAse I, 0.5 mg/ml lysozyme, 150 mM NaCl, 1 mM DTT, 1% NP-40, 1 × protease inhibitor), for homogenize and centrifugation. The supernatant was incubated with 100 ul of glutathione sepharose beads for 1 hour at 4°C. These purified proteins were subject to phosphatase assays using DiFMUP as substrate. To assay for enzymatic activity, RPTPγ cyto D1, RPTPγ cyto D1 C1061S or RPTPγ cyto D1 D1028A enzyme was incubated with 400 uM DiFMUP substrate in phosphatase assay buffer (25 mM MOPS pH 7.0, 50 mM NaCl, 0.5 mM EDTA, 1 mM DTT) in 96-well flat bottom black plate. 100 mM vanadate was used as inhibitor when needed. The reaction was carried out at room temperature and the conversion rate of DiFMUP substrate to highly fluorescent 4-Methylumbelliferyl was monitored in real time at excitation/emission wavelength 360/460 nm by TECAN fluorescence microplate reader. We confirmed that RPTPγ D1 C1061S mutant was completely inactive while D1028A retained about 1% of activity level of wildtype (data not shown).

Active phosphatase enzyme containing cytosolic region of RPTPγ-mychis was purified by Talon column as described [36], [37]. This enzyme was used to confirm that RPTPγ can dephosphorylate itself such as a phosphotyrosine containing RPTPγ, and a phospho-peptide spanning Y1307 derived from its own D2 domain. Phosphotyrosine containing His-tagged RPTPγ was immunoprecipitated by anti-his antibody and treated in 0.1% SDS to partially denature the protein. Then this protein was used as substrate for RPTPγ to confirm dephosphorylation. The phosphopeptide with >90% purity was synthesized by Sigma (Spring, Texas). Malachite green was used to monitor the release of phosphate from the reaction. Fourteen nanomolar cytosolic RPTPγ phosphatase was used in reaction and Km and Vmax were averaged from four independent phosphatase reactions.

## Results

### Phenotypic Testing

Starting at 9 weeks of age, wild type (WT) and mutant (MT) animals were analyzed using a standard phenotypic analysis battery used at Lexicon Pharmaceuticals (as an example, the phenotypic screen of VGLUT1 mice is accessible at: http://www.informatics.jax.org/external/ko/lexicon/2383.html), as previously described [31], [32]. The phenotypic analysis of both mutants (knockdown mutant mice and knockin mutant mice) reported here revealed no notable abnormalities in either gender across a wide range of behaviors, except in behavioral models sensitive to antidepressants.

### Knockdown Mutant Mice

#### Locomotor activity

There were no significant effect of sex or sex x genotype interaction, therefore data from males and females was combined for analysis. There was a significant effect of genotype with MT exhibiting higher locomotor activity than WT mice (2122±192 (10) for the WT and 3341±375 (10) for the MT, t(18) = 2.89, p<0.01).

#### Tail suspension (TS)

Two separate cohorts of mice were tested in the TS test, with the first cohort containing only female mice. In the first cohort there was a significant decrease in immobility time in the MT compared to WT (187.7±13.50 (8) for the WT, 106.2±21.15 (12) for the MT, t(18) = 2.87, p<0.05). In the second cohort, which contained both males and females evenly distributed among genotype, there was also a significant decrease in immobility time in the MT compared to WT mice (172.9 s ±13.53 (12) for the WT, 111.3 s ±13.84 (12) for the MT, t(22) = 3.18, p<0.01). There was no significant effect of sex or sex x genotype effect in the second cohort tested.

#### Locomotor activity and TS repeated 4-days testing

To evaluate effect of repeated exposure and habituation on behavior in the OF and TS tests and see if increased activity observed in the OF correlated with decreased immobility in the TS the same mice were run daily in the OFA in the morning, and in the TS in the afternoon. Analysis of total distance over days indicated a trend of genotype approaching significance [F(1,17) = 3.99, p = 0.06]. There was also a significant day x genotype interaction [F(3,51) = 4.90, p<0.05]. Analysis of interaction showed that total distance was significantly increased in MT mice on days 1 and 4, compared to the WT (Fig. 3A).

Analysis of immobility time in the TS over days indicated significant effect of genotype with MT mice having lower immobility than WT cohort-mates [F(1,17) = 23.7, p<0.001]. There was also a significant day x genotype interaction [F(3,45) = 3.24, p<0.05]. Analysis of the interaction showed that immobility in MT mice was significantly decreased on all test days, compared to the WT (Fig. 3B).

Correlation analysis showed that there was no significant correlation between immobility and activity in the MT mice on any of the testing days (all p-values >0.10). There was a significant correlation between immobility and activity in the WT mice only on the first day of testing (r = –0.65, p<0.05) and no significant correlation on other testing days.

#### Forced swim

There was no significant effect of sex or sex x genotype interaction for immobility, struggle and swimming on both pre-test and test days and therefore data from males and females was combined for analysis. There was no difference in struggling between genotypes on both days of FST. There was significant decrease in immobility in the MT on the pre-test day [F(1,18) = 20.3, p<0.001] and on the test day [F(1,18) = 21.6, p<0.001]. There was also significant increase in swimming on the pre-test day [F(1,18) = 5.78, p<0.05] and on the test day [F(1,18) = 16.3, p<0.001] in the MT mice, compared to WT cohort-mates (Fig. 3C).

### Knockin Mutant Mice

#### Locomotor activity

There was no difference in activity levels between WT and MT males (TD traveled in the OF test was 2632±297 (13) for the WT and 2706±281 (11) for the MT) or females (2921±250 (12) for the WT and 2526±249 (7) for the MT). There was also no difference in any other behavioral parameters measured in the OF test between the two genotypes.

#### Tail suspension

There was a significant effect of sex in the TS test with females of both genotypes having higher immobility than males [F(1,36) = 16.4, p<0.001]. When sexes were analyzed separately there was a significant decrease in immobility time in the male MT, compared to male WT (t(13) = 2.72, p<0.05), but no difference observed between MT and WT females (Fig. 4A).

#### Forced swim

There was no difference in immobility, struggling or swimming between genotypes on the pre-test day of FST. There was also no significant effect of sex or sex x genotype interaction for any of these parameters for pre-test and test days and therefore data from males and females was combined for analysis. On the test day there was a significant decrease in immobility [F(1,39) = 5.28, p<0.05] and increase in swimming [F(1,39) = 4.33, p<0.05] with an almost significant trend for increased struggle [F(1,39) = 3.76, p = 0.06] in the MT mice, compared to WT cohort-mates (Fig. 4B).

### Receptor Tyrosine Phosphatase Gamma (RPTPγ) Dephosphorylates Itself at Tyrosine 1307

Plasmids coding for wild type RPTPγ and “substrate trapping” mutant RPTPγ C1060S and RPTPγ D1028A were transfected to HEK293 cells and the proteins were immunoprecipitated (figure 5). RPTPγ C1060S was shown to be totally devoid of any phosphatase activity, and RPTPγ D1028A to have minimal activity at 1% of that of the wild type, when expressed in cytoplastic form on a generic phospho-substrate DiFMUP (see methods). We found that RPTPγ C1060S protein was in a tyrosine phosphorylated form reacted to antibody 4G10 in HEK293 cells, presumably by a tyrosine kinase yet to identify, and this phosphorylation was not present in wild type RPTPγ in the HEK293 cells (figure 5). RPTPγ D1060A also contained phosphotyrosine, though at a much weaker extent than that of RPTPγ C1060S. This result demonstrates that RPTPγ C1060S “trapped” the phosphorylated substrate which is itself and therefore suggests wild type RPTPγ dephosphorylates a phosphotyrosine in RPTPγ in HEK293 cells. Indeed in an in vitro experiment purified RPTPγ was able to dephosphorylate p-RPTPγ C1060S (figure 6).

A polypeptide that includes the site for this auto-dephosphorylation could serve as a substrate for assays to discover specific RPTPγ inhibitors. We then planned to map the amino acid site of phosphorylation in RPTPγ C1060S. First we constructed a truncation mutant of RPTPγ C1060S (aa. 1–1127) with D2 domain deleted. This D2 domain truncation abolished the presence of “self-trapped” phosphotyrosine (data not shown), suggesting that the dephosphorylation site lies within D2 domain or loss of that region causes significant structural changes. We then identified the 11 tyrosines that exist in the D2 domain (figure S1). We mutated singly each tyrosine to phenylalanine in the context of trapping mutant RPTPγ C1060S and performed transfection of each of 11 mutants to HEK293 cells. All mutants except RPTPγ C1060S/Y1307F contained tyrosine-phosphorylation that reacted to antibody 4G10 (figure 7). This result demonstrates that Y1307 of RPTPγ is the tyrosine residue that was phosphorylated, by activity of an unknown kinase, and then dephosphorylated by active RPTPγ.

Phosphotyrosine 1307 on RPTPγ is its first substrate site identified in living cells for phosphatase RPTPγ. We synthesized the peptide ATQDD(pY)VLEVR spanning Y1307. When this peptide was used as a substrate in a dephosphorylation reaction catalyzed by purified RPTPγ, we determined that Km was 53±5.0µM, Kcat was 2.8±0.4 s^−1^ and Kcat/Km was (5.2±0.3)×10^5−^M^−1^s^−1^ (figure 8).

## Discussion

RPTPγ is a transmembrane tyrosine phosphatase possessing multiple domains of uncharacterized functions. Previous studies by Lamprianou et al. [23] reported that RPTPγ gene ablation in mice produced a couple of modest phenotypes in neurological behavioral tests and a grossly normal brain development without any apparent anatomical abnormality. While RPTPγ is not required for gross brain development, the data from a loss of whole protein molecules do not address the potential for unique roles of different functional domains. In particular, RPTPγ might be functional in neuronal recognition/adhesion via its extracellular domains in addition to a separate role via tyrosine dephosphorylation by intracellular phosphatase domain. In present study, we create two novel genetic mutations of RPTPγ gene in mice to better understand RPTPγ functions in neurological behaviors. One mutant line is a RPTPγ gene “knockdown” in mice in which RPTPγ expression level is just about 1–2.5% of normal animals. The other mutant line contains a “knockin” point mutation on RPTPγ gene in mice which shows a loss of the phosphatase activity of RPRPγ. Our studies reveal that knockdown of RPTPγ gene in mice produces antidepressive-like behaviors as there is significantly decreased immobility time in the tail suspension test in mutant animals. The loss of phosphatase activity, shown by studies using genetic knockin mutant mice, also led to antidepressive-like phenotype as there is significantly decreased immobility time in the tail suspension test in the male mice and significantly decreased immobility time in the forced swim test. The phosphatase-inactive mutant lacking the enzymatic activity is a full length protein which should retain all other functions of RPTPγ such as interaction with the extracellular and intracellular components. We therefore conclude the loss of phosphatase activity is relevant to producing the antidepressive-like phenotype.

Interestingly, the near complete loss of the protein in the genetic knockdown, in addition to antidepressive-like behavior, also produced increased locomotor activity in knockdown animals shown by their significantly increased total distance traveled in open field test, which was absent in phosphatase-dead knockin mice. We hypothesize that the phosphatase activity and other functions of RPTPγ (such as cell surface recognition) differentially regulate neural functions that result in antidepressive-like and hyperactivity behavior in mice upon loss. Though it is plausible that increased locomotor activity in knockdown animals might be a confound to the interpretation of decreased immobility in tail suspension test, our additional behavioral studies indicate that decreased immobility in tail suspension and in the forced swim test can be separated from increased locomotor activity. In previous report by Lamprianou et al. [23], while the RPTPγ deficient mice did not have altered total time immobile in tail suspension, they had significantly increased latency to first immobilization suggesting an antidepressive like behaviors. Lamprianou et al. reported that their animals were generated using W4 ES cells (129S6/SVEvTac), and “wild-type and heterozygous or homozygous animals were either maintained as pure 129SvEv stock or used at a third-generation backcross to C57BL/6 for behavioral analysis” [23]. Thus Lamprianou et al. employed a different breeding strategy and therefore the background of mice was not the same as ours which could explain some of the phenotypic differences.

The decreased immobility time in the tail suspension of knockin mice is only observed in males but not in the females when compared to the gender-matched controls. One possibility is that the antidepressive phenotype in the knockin animals is not as robust as in the knockdown animals. While we conclude the loss of phosphatase activity is relevant in producing antidepressive effects, we cannot exclude the possibility that the “docking” capacity of the whole protein, upon loss, may partially contribute to production of antidepressive-like behavior. Additionally, it is well recognized that the gender-specific factors may influence significantly the behaviors in mice. Ptprg gene may have specific implication in the estrogen pathway, as it was reported that Ptprg gene expression is regulated by 17-beta-estrodial via the estrogen receptor in cultured cells [38].

In biochemical study, we discover RPTPγ autodephosphorylates itself in HEK293 cell line and identify residue Y1307 at D2 domain of RPTPγ as a site of dephosphorylation. Protein tyrosine phosphatases tend to have little specificity in vitro towards substrates. Particularly, it was reported that RPTPγ was active on many phospho-peptides in the in vitro enzymatic reactions [39] and was one of the most promiscuous phosphatases among 25 different phosphatases tested [39]. This implies that the specificity for substrates by RPTPγ is achieved via regulatory mechanisms present in vivo. Our discovery of Y1307 in the D2 domain as a substrate of RPTPγ is based on a substrate-trapping in the living cultured cells, which may be similar to endogenous condition. The dephosphorylation was confirmed in vitro using phospho-Y1307 containing peptide as substrate. We found that the Km of the dephosphorylation of phospho-Y1307 containing peptide by RPTPγ falls in the physiological concentration range. An in vivo assay system would be desired to directly confirm the dephosphorylation event on Y1307 under endogenous condition and this will be the direction of future research. The future study of autodephosphorylation at Y1307 will evaluate its potential usefulness as native substrate for inhibitor screen.

Tyrosine phosphorylation of PTPs suggests the intriguing possibility for autoregulation or feedback regulatory mechanisms. RPTPα is known to be phosphorylated on a C-terminal tyrosine. This phosphorylation site is a consensus GRB2-binding site and GRB2 binds readily to phosphorylated RPTPα [40], [41]. It has also been suggested that RPTPα can undergo autodephosphorylation [40]. CD45 was found to be phosphorylated transiently on tyrosine and its phosphatase activities are enhanced by this phosphorylation [42], [43]. Autodephosphorylation of Y1307 of RPTPγ possibly plays a role in regulating the signaling activity of this receptor tyrosine phosphatase. Additional investigation of RPTPγ activity and regulation may lead to a better understanding of the biochemical underpinnings of human depression.

## Supporting Information

Figure S1 Site-directed mutagenesis to map RPTPγ auto-dephosphorylation site- Eleven tyrosine residues in RPTPγ’s D2 domain were bold and underlined, as potential autodephosphorylation site. Each tyrosine was mutated phenoalanine by site-directed mutagenesis.
(TIF)
